# Role of Innate T Cells in Anti-Bacterial Immunity

**DOI:** 10.3389/fimmu.2015.00302

**Published:** 2015-06-11

**Authors:** Yifang Gao, Anthony P. Williams

**Affiliations:** ^1^Academic Unit of Cancer Sciences, Faculty of Medicine and Institute for Life Sciences, University of Southampton and NIHR Cancer Research UK Experimental Cancer Medicine Centre, Southampton, UK; ^2^Wessex Investigational Sciences Hub (WISH) Laboratory, Department of Allergy, Asthma and Clinical Immunology, University Hospital Southampton NHS Foundation Trust, Southampton, UK

**Keywords:** iNKT cells, MAIT cells, gamma delta T cells, innate T cells, bacterial infections

## Abstract

Innate T cells are a heterogeneous group of αβ and γδ T cells that respond rapidly (<2 h) upon activation. These innate T cells also share a non MHC class I or II restriction requirement for antigen recognition. Three major populations within the innate T cell group are recognized, namely, invariant NKT cells, mucosal associated invariant T cells, and gamma delta T cells. These cells recognize foreign/self-lipid presented by non-classical MHC molecules, such as CD1d, MR1, and CD1a. They are activated during the early stages of bacterial infection and act as a bridge between the innate and adaptive immune systems. In this review, we focus on the functional properties of these three innate T cell populations and how they are purposed for antimicrobial defense. Furthermore, we address the mechanisms through which their effector functions are targeted for bacterial control and compare this in human and murine systems. Lastly, we speculate on future roles of these cell types in therapeutic settings such as vaccination.

A successful immune response to foreign pathogen requires a rapid activation of innate immunity, which directs the subsequent development of a productive adaptive immune response. Innate T cells represent a group of T lymphocytes that are able to act during the lag time while effective adaptive immune responses develop (1). Similar to conventional T cells, innate T cells undergo T cell receptor (TCR) rearrangement and thymic selection. Unlike their conventional counterparts, innate T cells rapidly recognize foreign pathogen signals and manifest immediate effector functions after activation. This allows innate T cells to perform effector immune responses much earlier than conventional T cells, and act as an additional “bridge” between innate and adaptive immune responses (2).

Classically, T cells are subdivided into two major populations based upon their TCR expression, namely alpha beta (αβ) T cells and gamma delta (γδ) T cells. Conventional αβ T cells recognize a broad range of peptide antigens typically presented by Major Histocompatibility Complex (MHC I and II) complexes, enabled through their highly diverse TCR arrangement. In contrast, the αβ innate T cells that have been identified display a restricted T cell repertoire characterized by the expression of an invariant or semi-invariant TCR α chain. In humans, two well-defined αβ innate T cell populations have been identified in recent years, namely, mucosal-associated invariant T (MAIT) cells and invariant natural killer T (iNKT) cells. These two T cell populations together with γδ T cells form the three major types of innate T cell (1). All three innate T cell populations express a C-type lectin molecule CD161. CD161 was initially identified on CD4, CD8, and γδ subsets in the 1990s (3, 4). CD161 is variably expressed across human T cells, and three populations can be identified, expressing negative, intermediate, and high levels of CD161 (2). The expression of CD161 in human T cells populations is summarized in Figure 1. The level of CD161 expression is distinctive between conventional T cells and innate T cells, with MAIT cells displaying the highest levels (5).

**Figure 1:**
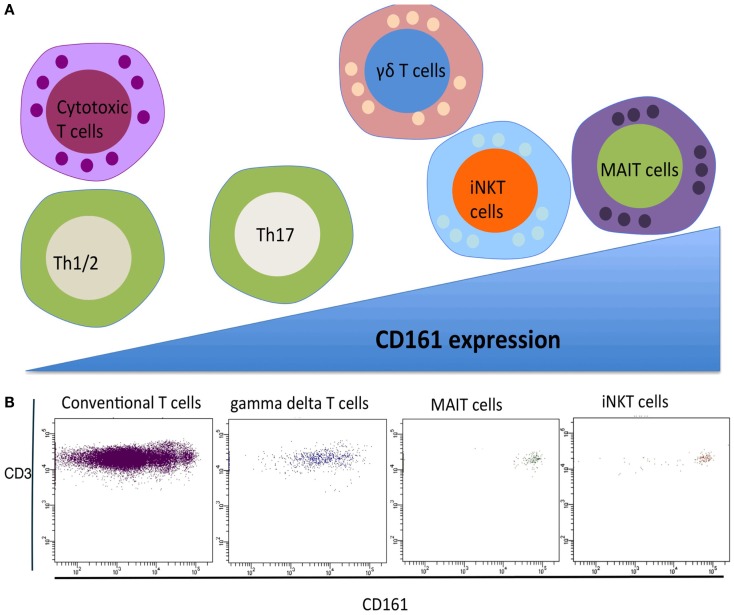
**(A)** CD161 expression of T cell subsets. Gamma delta T cells, iNKT cells, and MAIT cells express CD161 at a higher level compared to conventional T helper and T cytotoxic T cells. **(B)** FACS analysis of CD161 expressing in different human T cell subsets.

While innate T cells are distinctive as a subpopulation of T cells, they have other distinct features, which do not overlap. They share the absence of MHC I/II peptide restriction but differ in their respective antigen presentation modalities. iNKT and MAIT cells respond to the MHC-like molecules, CD1d and MR1, respectively, while CD1c can present antigens to γδ T cells. The nature of the antigens recognized by innate T cells is also diverse and broadly non-overlapping involving metabolites, bacterial products, and lipids. iNKT cells have been principally shown to respond to glycolipids, γδ T cells are potently activated by (E)-4-hydroxy-3-methyl-but-2-enyl pyrophosphate (HMBPP), and MAIT cells can be activated by riboflavin metabolites – reduced 6-hydroxy methyl-8-d-ribitylumazine (rRL-6-CH_2_OH), as well as folic acid metabolite, 6-formyl pterin (6FP). Finally, the sites of development, residence, and frequency within the T cell poll are distinctive and summarized in Table 1.

**Table 1:** **Characteristics of innate T cells**.

TCR	Human: Vα24-Jα18	Human: Vα7.2-Jα33	Human: Vδ1, Vδ3, Vγ9vδ2
	Mouse: Vαl4-Jα18	Mouse: Vαl9-Jα33	Mouse: VγlVδ6.3, Vγ5Vδ1, Vγ6Vδ1
Ligand	Glycolipids, phospholipids	Vitamin B2 metabolites, transitory neo-antigens	Phosphoantigens, phycoerythrin, glycolipids
Frequency	Low (0.01–1% of T cells)	1–20% of T cells	2–10% of T cells
Location	Blood, mucosal site, and liver	Blood, gut, lung, liver	Blood, mucosal sites
Maturation	Thymus	Secondary lymphoid tissue	Thymus
Present at birth	Yes	Yes	Yes
HSCT	Yes	?	Poor

*?, information not known*.

## iNKT Cells

Invariant NKT cells are one of the most well-studied innate T cell populations (6–8). These cells are defined by their semi variant TCR, CD1d antigen restriction, and glycolipid recognition. Numerous studies have been undertaken with these cells following the discovery of their specific ligand, alpha-galactosylceramide, in 1997 (7, 9–11). Over the subsequent years, a range of endogenous and exogenous lipid antigens has been identified, which may change the effector responses of this innate T cell population (12–16). This cell population is also notable for its expression of previously considered NK cell specific markers such as CD161, which has subsequently been recognized on other innate T cell populations (17).

Invariant NKT cells develop in the thymus and are present at a very low number in most tissues. They are selected by CD1d, which is expressed on double-positive (CD4, CD8) thymocytes, through the recognition of endogenous lipids. In the thymus, iNKT cells acquire a memory/effector phenotype prior to exiting to the circulation. Recent studies have suggested that post-thymic education is required for iNKT cells to become fully mature and achieve functional competency (18, 19). In human peripheral blood, approximately 0.01–1% of the T lymphocytes are iNKT cells, characterized by their hallmark TCR-invariant chain Vα24-Jα18 and variant Vβ11; and Vα14-Jα18 in mice with a limited number of β chains, including Vβ8.2, Vβ7, and Vβ2 (20). Despite their presence in relatively low numbers in humans, iNKT cells can be very effective in early host defense mechanisms and are involved in a variety of disease settings (6, 21–23). A key feature of iNKT cells is their rapid release of a wide array of cytokines and chemokine following ligand activation (17). This plays an important role in their early effector and regulatory properties. Our understanding of the importance of iNKT cells is largely based on disease studies undertaken in iNKT deficient mice (24). Previous studies have shown that iNKT cells play an important role in the detection of various pathogens, including *Pseudomonas aeruginosa*, *Streptococcus pneumoniae*, *Salmonella typhimurium, Mycobacterium tuberculosis*, *Listeria monocytogenes*, and *Borrelia burgdorferi* (1, 25, 26). In addition to bacterial infections, iNKT cells have also been found to play an important role in viral infections, including influenza, cytomegalovirus, and coxsackie B3 viral diseases (25, 27). Finally, they also play an important role in tumor immunity (28) and autoimmune disease (22). In human studies, a link between defects in iNKT cells may lead to susceptibility to certain infectious diseases, such as tuberculosis (29, 30), EBV (31–33), allergy (34), atherosclerosis (35), and immunodeficiency. (10, 36–38).

### Role during bacterial infections

The presence of functional iNKT cells during bacterial sepsis has been shown in a number of different murine settings. In *S. pneumoniae* infection, a much higher level of bacteria were identified in the Jα18 knockout mice compared to the iNKT competent wild type mice, resulting in significant survival rate differences between the two strains (39). In the iNKT knockout mice, a defect was found in neutrophil recruitment to the lung together with a reduced production of neutrophil chemo-attractants, including TNF-alpha and MIP-2. A reconstitution of iNKT cells from wild-type mice to iNKT deficient mice was able to restore the production of TNF-alpha and MIP-2, leading to improved neutrophil and bacterial clearance (40). In a further bacterial infection caused by *Chlamydia pneumoniae*, accumulations of iNKT cells within the lung were visible within hours of acute infection, demonstrating IFN-gamma production at the site of infection (41). An extension of conventional bacterial challenge studies has recently been undertaken by Wong et al., which suggested that iNKT cells might play a role in control of bacterial infections associated with stoke. Compared to their WT little mate, iNKT deficient mice were found to be more susceptible to bacterial infection post transient midcerebral artery occlusion. This was related to the ability of iNKT cells to act as a suppressor for neurotransmitter release post-stroke, which is lost in iNKT deficient mice, making them more susceptible to the bacterial infection (42). In humans, several studies have established the link between iNKT cells and *M. tuberculosis* infection with both the function and number of iNKT cells reduced in these patients (43). Two distinct pathways have been proposed for iNKT cell activation during infection. They can either be directly activated through TCR-CD1d-glycoplid recognition or indirectly through their response to innate cytokines that are released from other innate cells.

### Indirect activation of iNKT cells by gram-negative bacteria

Early secretion of IFN-gamma can be induced by iNKT cells following an encounter with both Gram-negative and Gram-positive bacteria. Innate receptors that recognize bacterial signals have a crucial role in triggering the antigen presenting cells, which subsequently direct the activation of iNKT cells (9, 44–49). The activated antigen presenting cells stimulate the iNKT cells by signaling through toll like receptors (i.e., TLR4, TLR7, and TLR9) leading to the production of IL-12, also other inflammatory cytokines. Studies by De Libero and Paget have suggested that TLR signaling through APCs are not only important for cytokine production but also the accumulation of self-lipid antigen for CD1d presentation (47, 50). A study by Darmoise et al. showed that the TLR signaling triggered the accumulation of self-lipid including iGb3 in the lysosome, leading to an enhanced iNKT cell activation (51).

### Direct activation of iNKT cells by gram-negative bacteria

Another mechanism that allows iNKT cells to respond to bacterial infection occurs through the direct recognition of the glycosphingolipid in the cell wall of Gram-negative bacteria. One such example is *Sphingomonas/Novosphingobium spp*., where the glycosphingolipids present in the bacteria cell wall are alphagalacturonylceramides and alphaglucuronylceramides (52). These glycosphingolipids contain one sugar ring and have been showed to activate iNKT cells *in vitro*, while multi-sugar ring glycosphingolipids have not been able to activate iNKT cells in co-culture. Murine studies suggested that CD1dKO mice were able to clear infections with *Sphingomonas/Novosphingobium* as well as some other LPS-negative bacteria, but at a much slower rate compared to the wild type mice (45, 53, 54). This would suggest the iNKT cells are one of the major innate cell types involved in bacterial clearance and playing a major role in the early response.

## γδ T Cells

γδ T cells are another group of innate T cells that have been found to play an important role during bacterial infections. Unlike conventional αβ T cells, γδ T cells do not usually express a CD4 or CD8 lineage marker and they do not require conventional antigen presentation via MHC class molecules (55). Different subtypes of γδ T cells have been described often identified by the different arrangement of their TCRs in early development. The differences in TCR arrangement directly influence their eventual principle tissue of residence. In human, the majority of the γδ T cells present in the peripheral blood express the TCR Vγ9Vδ2, whereas Vδ1 and Vδ3 TCR are primarily expressed at the mucosal surfaces. In mice, Vγ1 and Vγ4 are present in the lymphoid tissues; Vγ5 is found to be present in the skin; Vγ6 in the reproductive tract; and Vγ1, Vγ4, and Vγ6 present in the lung (56). A number of mechanisms have been described linking γδ cells and bacterial infections. Similar to iNKT cells, γδ are able to sense danger signals in both a TCR dependent and TCR independent way. γδ T cells can be activated by Class I like molecules such as T10/T22 (in mice) and members of CD1 family; they can also be activated by MHC-unrelated molecules such as viral glycoproteins and F1-ATPase complex in human (57–59). In addition to TCR recognition, γδ T cells also express pattern recognition receptor and receptor typically associated with NK cells.

γδ T cells may expand in the patient’s peripheral blood during bacterial infections with studies identifying up to 12% in listeriosis, 14% in tuberculosis, and 29% in brucellosis (60). Human γδ T cells respond to bacterial infections by recognizing (E)-4-hydroxy-3-methyl-but-2enyl pyrophosphate (HMBPP) derived from various bacteria. γδ T cells were shown to be particularly important in response to intracellular bacterial pathogens including *M. tuberculosis* and *Legionella micdadei*. In the case of *L. micdadei*, Vγ9Vδ2 T cells were found to be depleted from the circulation upon bacterial infection, followed by a sharp increase, then a slow decline over a 6-month-period (61). This dynamic change may indicate Vγ9Vδ2 might be important in contributing to the pathophysiological changes of Pontiac fever-like disease. A similar kinetic pattern is seen with *M. tuberculosis* infection, following the Vγ9Vδ2 T cell response to the metabolite IPP (62).

Early studies found that Vγ9Vδ2 T cells were the most important group that led to the eradication of bacteria (63, 64). Seminal studies identified the antigens involved in the recognition were intermediates in isoprenoid biosynthesis, namely (E)-4-hydroxy-3-methyl-but-2enyl pyrophsphate (HMBPP) (65). The level of HMBPP directly influences the magnitude of Vγ9Vδ2 T cell activation and proliferation (66). Recently, a major breakthrough in discovering the mechanisms for activating the Vγ9Vδ2 T cells was made by Bonneville and Scotet’s group. They identified that a member of butyrophilin molecule family CD277 played a crucial role during γδ T cells activation (67–71).

γδ T cells have also been found to be able to promote self-activation through cell to cell interaction (72). However, it was demonstrated that the self-activation mechanism is not as effective as formal presentation through antigen presenting cells (73–75). One important aspect of γδ T cells is that they can trigger the maturation of dendritic cells. Devilder et al. showed that Vγ9Vδ2 T cells can stimulate the maturation signal on mycobacterial infected DCs, through a Fas–Fas ligand interaction (76) and/or TCR-CD1 contact (77). Other than dendritic cells, γδ T cells have also been found be important in macrophage recruitment. During infection with *listeriosis*, γδ T cells were found to be a key player in controlling the production of key macrophage chemo attractants (78). Skeen et al. also showed that macrophages failed to undergo maturation in the absence of γδ T cells (79). Direct engagement of γδ T cells may facilitate pathogen clearance through their production of bacteriostatic and lytic molecules, such as granulysin and defensins. During *Staphylococcus aureus* respiratory infection, γδ T cells sense the dysregulation of the mevalonate pathway within the infected cells. This leads to the activation and expansion of γδ T cells, in particular, Vγ9Vδ2 T cells. The active γδ T cells then produce cytokines such as IL-17, which leads to airway protection. γδ T cells also play a role during *M. tuberculosis* infection, producing a variety of cytokines including IFN- γ, TNF-α, and IL-17. IFN-γ and TNF-α play are essential in host protection against *M. tuberculosis* enabling granuloma formation and disease containment.

## MAIT Cells

Mucosal-associated invariant T cells are the newest members of the innate T cell family. They were first described by Tilloy et al. (80) and represent the most abundant innate T cells in humans. They express a canonical Va7.2-Ja33 chain in humans and the orthologous Va19-Ja33 in mice. The development of MAIT cells is parallel to the development of iNKT cells and both express the transcription factor ZBTB16 (81). In adults, they display an effector phenotype, whereas MAIT cells possess a naïve phenotype in cord blood. In both cord and adult blood, MAIT cells express CD161, IL-18Ra, CCR6, and about 50% of the MAIT cells express the T cells co-receptor CD8 (82–84). Recent studies also show that MAIT cells express the ABC binding cassette (ABC) B1 drug resistance transporter (85, 86). MAIT cells have a further unique antigen recognition system recognizing a MHC Class I related molecule (MR1), which is able to present bacterial derived ligand. Study by Kjer-Nielsen et al. showed that 6-formyl pterin (6-FP), a metabolite on the folic acid pathway, could stabilize the MR1 molecule but failed to activate the cells. Full activation of primary MAIT cells was achieved with ligand reduced 6-hydroxymethyl-8-d-ribityllumazine (rRL-6-CH_2_OH), a riboflavin metabolite. Related products 7-hydroxy-6-methyl-8-d-ribityllumazine (RL-6-Me-7-OH) and 6,7-dimethyl-8-d-ribityllumazine (RL-6.7-diMe) have also shown similar agonistic activity for MAIT cells, leading to the rapid production of cytokines (87). In recent years, studies on MAIT cells have associated their number and function with diverse of disease settings, including bacterial infections and autoimmune disorder.

The first hint that MAIT cells have anti-bacterial activities was described in 2010, where studies by Gold et al. and Le Bourhis et al. showed that MAIT cells could recognize a range of bacteria species through MR1 (88, 89). In the study by Gold et al., MAIT cells could respond to *M. tuberculosis* even in unexposed individuals. They further showed that MAIT cells responded to *Salmonella enteria*, *Escheichia coli*, and *S. aureus* infected APC (90). Le Bourhis et al. showed that MAIT cells could MAIT cells are able to respond to a wide array of bacteria including Gram-positive Bacteria *S. aureus*, *Staphylococcus epidermidis, Lactobacillus acidophilus*, and Gram-negative Bacteria *E.coli, Klebsiella pneumonia, Pseudomonas aeruginosa*, and *Mycobacterium abscessus*. Importantly, some bacterial species were shown not to activate MAIT cells, namely *Enterococcus faecalis* and *Streptococcus pyogenes*, suggesting a novel specificity. The importance of their role in bacterial defense was suggested by a study by Georgel et al. demonstrating that during the *Klebsiella pneumonia* infection, MR1 deficient mice succumbed to disseminated infection whereas the WT mice achieved full bacteria clearance within 2 days (91). Similarly, in the Va19 transgenic mice, enhanced control of the *E.coli* and *M. abscessus* infection were observed. Further studies performed by Chua et al. and Meierovics et al. also showed that MAIT cells were needed in the early control of *Mycobacterium bovis*, BCG, and *Francisella tularensis* infection (92, 93). In humans, how MAIT cells play a role in infectious disease is less well understood. A number of studies have associated the frequency of MAIT cells in different infectious diseases (94). MAIT cell numbers were found to be lower in peripheral blood of patients with *M. tuberculosis* infection (95). Also, in a study of critically ill septic and non-septic patients, the patients with severe bacterial infections, but not viral infections, had a much lower MAIT count compared to healthy controls (96).

One of the most well-studied examples of MAIT cells in bacterial infection is during *Salmonella* infection (97, 98). Upon activation, MAIT cells produce IFN-gamma, TNF α, and IL-17. These cytokines have been shown to be critical in controlling *Salmonella* infections, with IL-17 preventing the dissemination of infection (99). MAIT cells may also play a role during *Salmonella* infection through their early cytotoxic activity (100), although further studies are needed as MAIT cells were not able to directly kill *Salmonella* infected cell lines (94, 101).

**Figure 2:**
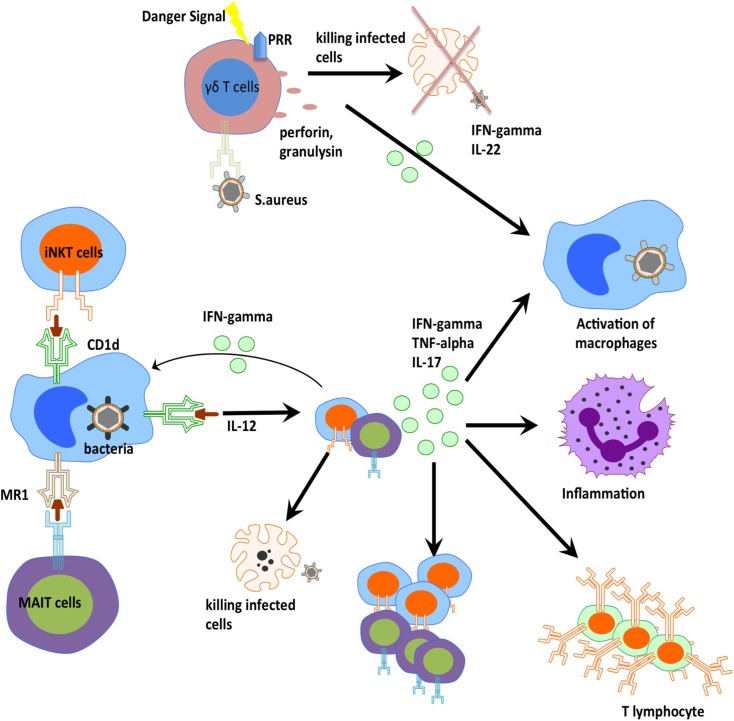
**Orchestration of innate T cells in anti-bacterial immunity**. iNKT cells, MAIT cells, and gamma delta T cells play an important role in anti-bacterial defense through cytokine production, perforin release, and cross-talk to other innate and adaptive cells.

Over the last 5–10 years, there has been advancement in the understanding and description of unconventional T cells. These studies demonstrate that unconventional T cells do indeed play an important role during bacteria infection and contribute the ability of host organism to clear and control certain bacterial infections (Figure 2). These cells are able to efficiently traffic to the sites of inflammation, and initiate rapid responses by means of cytokine production and cytotoxic activities. Further studies will elucidate the molecular details of this cellular control suggesting novel approaches to how we may harness these cells through therapeutic vaccination and pharmaceutical manipulations.

## Conflict of Interest Statement

The authors declare that the research was conducted in the absence of any commercial or financial relationships that could be construed as a potential conflict of interest.
